# Host Plant Use by the Invasive *Halyomorpha halys* (Stål) on Woody Ornamental Trees and Shrubs

**DOI:** 10.1371/journal.pone.0149975

**Published:** 2016-02-23

**Authors:** Erik J. Bergmann, P. Dilip Venugopal, Holly M. Martinson, Michael J. Raupp, Paula M. Shrewsbury

**Affiliations:** Department of Entomology, University of Maryland, College Park, Maryland, United States of America; United States Department of Agriculture, Beltsville Agricultural Research Center, UNITED STATES

## Abstract

The brown marmorated stink bug, *Halyomorpha halys* (Stål) (Hemiptera: Pentatomidae) is an invasive plant-feeding insect native to eastern Asia. This herbivore is highly polyphagous, feeding on and damaging diverse plants, including field crops, vegetables, tree fruits, and ornamentals. Woody ornamental plants provide early- and late-season resources for adults emerging from and returning to overwintering sites, as well as feeding and breeding sites for *H*. *halys* throughout the growing season. In this study, we quantify the use of diverse plants by *H*. *halys* in two commercial nurseries in Maryland, recording data on the abundance of egg masses, early and late instar nymphs, and adults over a three-year study period. Our specific goals were to provide a quantitative comparison of the use of diverse plant species and cultivated varieties, identify non-hosts that could be used to create landscapes refractory to *H*. *halys*, and determine whether the use of plants varied across life stages of *H*. *halys* or the taxonomic status of plants. We found broad use of diverse plants in this study, identifying 88 host plants used by all life stages of *H*. *halys*. We also highlight the 43 plant taxa that did not support any life stage of *H*. *halys* and are thus classified as non-hosts. Interestingly, some of these plants were congeners of highly-used plants, underscoring high intrageneric and intraspecific variation in the use of plants by this polyphagous herbivore. We discuss how the selective planting of non-hosts, especially gymnosperms, may aid in reducing the agricultural and nuisance pest status of this invasive insect.

## Introduction

The brown marmorated stink bug, *Halyomorpha halys* (Stål) (Hemiptera: Pentatomidae), an insect native to Japan, China, Taiwan, and Korea, was first discovered in the United States near Allentown, PA in the middle1990’s [1]. *H*. *halys* is currently reported in 42 states, the District of Columbia, and two Canadian provinces in North America [2]. Beyond North America, *H*. *halys* has invaded several European countries including Lichtenstein and Switzerland [3], France [4], Italy [5], Germany [6], Hungary [7] and Greece [8]. Interceptions in Australia [9] and New Zealand [10] are also becoming increasingly frequent.

*H*. *halys* causes direct damage to plants, importantly to many commercial crops, through its feeding activities [11–18] and can cause indirect damage through the transmission of plant diseases including Paulownia witches’ broom [19]. During the growing season of 2010, populations of *H*. *halys* burgeoned in the United States, and multimillion dollar losses were recorded on orchard crops including apples and peaches; vegetables such as sweet corn, peppers, and tomatoes; row crops including field corn and soybeans; vineyards; small fruit; and ornamental plants grown in landscapes and nurseries [14,15,20]. In addition to crop damage, *H*. *halys* is a severe nuisance pest during fall, winter, and spring when adults aggregate on commercial buildings and homes, enter and overwinter in domiciles, and egress in spring [15,21–25]. These behaviors generate public concern, media attention, and a general outcry for management solutions [15,23,24]. In response to this demand, pest control companies provide services including the treatment of buildings and landscape plants where *H*. *halys* aggregate in autumn prior to entering structures [25].

*Halyomorpha halys* is highly polyphagous in both its native and invaded ranges. An important review of the Asian literature revealed 106 hosts distributed in 45 families, ranging from herbaceous annual vegetable crops to forest trees [12]. A synthesis of host use [24] reported 51 host plants in 32 plant families in Europe. This list included European native and non-native plants, ranging from herbaceous perennials to woody trees and shrubs. In the invaded North American realm, studies conducted in several counties in eastern Pennsylvania recorded *H*. *halys* on 73 species of plants ranging from annual crops to landscape trees [21]. Trees and shrubs, many of which were not native to North America, dominated the list of plants upon which *H*. *halys* was noted as abundant or common [21]. The greatest numbers of *H*. *halys* were found on tree of heaven, catalpa, yellowwood, paulownia, cherry, walnut, and redbud growing in non-managed woodlands in North Carolina and Virginia [26]. Additional investigations of ornamental and cultivated hosts have demonstrated temporal and stage specific shifts in host use of *H*. *halys* over the course of growing seasons [11]. Because of the use of plants of non-Asian origin in both Europe and North America, it is apparent that the host plant list from Asia will be an incomplete list of the plants used by *H*. *halys* in its invaded range.

The use of resistant plant material is a mainstay of integrated pest management for agronomic crops [26,27] as well as for ornamental plants in landscapes [28–30]. Several of the aforementioned reviews of *H*. *halys* noted significant variation in patterns of host use among woody landscape plants. However, these reviews focused on plants on which *H*. *halys* was observed feeding or breeding; with the exception of the survey by Bakken et al. [31], little or no information was presented on the plants that were not used as hosts by *H*. *halys*. Furthermore, although several Asian studies and reviews [12,24] noted *H*. *halys* utilizing many species of gymnosperms, gymnosperms have been conspicuously lacking in host lists from North America [11,21]. The extent of intraspecific variation in host plant use is also not well understood. Whereas previous studies have noted significant variation in the abundance of *H*. *halys* among varieties of tree fruits [32–34], little is known about intraspecific variation in host use among ornamental trees. Such information would be invaluable for developing surveys and management plans, and for identifying resistant plant material.

In this study, we examined patterns of host plant use by *H*. *halys* in large, diverse, commercial production nurseries in Maryland. Our specific goals were to quantify variation in the use of plants across life stages of *H*. *halys*, expand the host plant list by examining a broad diversity of plants, especially those that had not yet been surveyed for *H*. *halys*, and identify plants not used by any life stage, particularly ovipositing females. We also included gymnosperms and intraspecific variation in the plants surveyed, in order to develop a robust understanding of the variation in host plant use of *H*. *halys* in its invaded range.

Our study is novel in seeking to identify ornamental woody plants that are not included in the feeding or breeding repertoire of *H*. *halys*. By incorporating plants not used by *H*. *halys* into landscapes, we hope to obviate breeding sites and places where stink bugs aggregate prior to entering homes. This should reduce the need for treating structures and plants with insecticides to kill this pest [25]. Moreover, by identifying ornamental plants refractory to this pest, commercial growers of landscape plants could enjoy a marketing advantage by producing and selling plants that reduce the likelihood of autumnal home invasions. Commercial nurseries in this study provided a rich source of familial, generic, specific, and varietal variation in which to explore patterns of host use by *H*. *halys* on angiosperms and gymnosperms grown for planting in residential landscapes.

## Materials and Methods

### Ethics Statement

No endangered or protected species were involved in the study. We obtained permission from individual private commercial tree nurseries for access and data collection.

### Study design and data collection

We sampled for *H*. *halys* during a three-year period in two commercial woody plant nurseries located in Frederick and Montgomery Counties, Maryland. We recorded *H*. *halys* life stages through timed visual surveys in each nursery on trees and shrubs on multiple occasions each year. Surveys conducted in 2011 occurred at several production fields at Raemelton Farm in Adamstown in western Maryland (39.299468° N; 77.458549° W). In 2012 and 2013, surveys were conducted at several production fields at Raemelton Farm and at Ruppert Nurseries in Laytonsville in central Maryland (39.243172° N, 77.142069° W). Production fields at Ruppert Nurseries consisted of 20 rows of 25–35 individual plants. Fields at Raemelton were larger and consisted of 80–150 rows. Rows at both locations were spaced approximately 3 m apart, and depending on the size of the plant, plants within rows were approximately 2 m apart. Plants ranged in height from 1 to 4 m. Typically, a single species or cultivar was grown per row, with extensive variation in tree identity between rows and fields in each nursery.

Following the protocols of Venugopal et al. [35] and Martinson et al. [18], 1-min visual counts of *H*. *halys* were conducted per plant, encompassing the foliage, flowers, fruits/seeds, and bark to a height of up to 3 m on six trees per row. Undergraduate and graduate student observers were trained by E.J.B. and M.J.R. to ensure uniformity and consistency in the field protocols for data collection. *H*. *halys* abundance (total per plant) was recorded separately for four life stages: egg masses, early instar nymphs (instars 1–3), late instar nymphs (instars 4 and 5), and adults. Each year, we conducted repeated surveys for *H*. *halys* on each tree from late May to early August: in 2011, each tree was surveyed four times; in 2012 and 2013, each tree was surveyed six times. Sale of some of the sampled trees during the study period, as well as tree mortality from heat stress, disease, and physical damage, resulted in variable numbers of surveys, or ‘tree visits,’ for some trees (see S1 Table for number of visits for each plant taxon).

### Tree identification

We recorded the genus, species, and cultivar (cultivated variety) of each tree. Trees without cultivar names are straight species, which have not been selected for particular traits. We completed the tree identification using nursery records and confirmed it using existing literature [36] to ensure consistent usage of scientific and cultivar names, common names, and spellings. We also ensured accuracy in nomenclature and taxonomy of the tree species and cultivars (hereafter, plant taxa) by comparing our records with The Plant List [37].

### Statistical analysis

#### Host use by different life stages

Definitions of ‘host’ are highly variable across studies, and the concept of host has been used in several contexts. With respect to *H*. *halys*, Oda et al. [38,39] (reported in [12]) described breeding plants (those on which eggs, nymphs, and adults were observed) and feeding plants (those on which only adults were observed feeding). In North America, Nielsen and Hamilton [11] classified plants as hosts based upon consecutive observations of nymphal stages across multiple years. Because plants supporting all life stages are suitable for adult oviposition as well as nymphal development, these plants represent reproductive hosts for *H*. *halys* [40,41]. Following this delineation, we classify plant taxa with records of each of the four life stages (egg masses, early nymphs, late nymphs, and adults) of *H*. *halys* as hosts. On the other hand, we classify plant taxa without any of the *H*. *halys* life stages as non-hosts. Plants with some but not all of the life stages are classified as partial hosts.

To test whether the use of plants was similar across all life stages of *H*. *halys*, we calculated the proportion of plant taxa used by each life stage. We used Person’s Chi-squared tests for pairwise (each life stage) statistical comparisons of these proportions.

We further ranked the plants classified as hosts based on mean abundance of *H*. *halys*, calculated as the summed abundance of *H*. *halys* nymphs and adults divided by the total number of 1 min. surveys conducted for that taxon.

#### Use of angiosperms and gymnosperms

The use of angiosperms and gymnosperms by *H*. *halys* was analysed through generalized linear models (GLM) assuming a Quasi-Poisson error distribution and log link function [42]. GLMs were performed for each life stage with the abundance of *H*. *halys* as the response variable and taxonomy as the predictor, accounting for differences in tree visits across the cultivars (through ‘offset’ statement). Significant differences in the model estimated means were identified through Tukey’s HSD comparisons (α = 0.05).

All statistical analyses were performed in the program R [43] and associated statistical packages. Nomenclature and other taxonomic details were verified using package ‘Taxonstand’ [44]. Tukey’s HSD were performed with the package “multcomp” [45]. Coefficients estimated by the GLMs were extracted and plotted using “ggplot2” [46].

## Results

### Host use by different life stages

Our surveys for *H*. *halys* encompassed 254 unique taxa of ornamental trees and shrubs. Over the three year study period, we conducted 52,717 one minute surveys, recording a total of 589 egg masses, 23,697 early instar nymphs, 3,525 late instar nymphs, and 10,925 adults. At least one life stage of *H*. *halys* was present on 211 (83%) of the surveyed plant taxa. Egg masses were present on 99 (39.0%), early nymphs on 176 (69.3%), late nymphs on 144 (56.7%), and adults on 198 (78.0%) taxa.

Host plant use differed among life stages, with a general increase in the proportion of available plant taxa utilized with increasing insect developmental stage. Specifically, adults were found on a higher proportion of plant taxa than were late nymphs and egg masses, and late nymphs were found on a higher proportion of plant taxa than were egg masses (Table 1). Early nymphs, on the other hand, used nearly as many plant taxa as adults and more than late nymphs. There were no cultivars on which only the egg masses, but no other stages, were recorded (see S1 Table). Despite differences in the proportion of plant taxa used by each life stage, broad patterns of overlap in plant use were also apparent across life stages (Table 1).

**Table 1:** Comparison of the proportions of plant taxa used between pairs of life stages of *Halyomorpha halys*.

Life stage comparison	χ^2^ value	P-value ^*a*^	% of all plant taxa shared
Egg masses–Early nymphs	45.79	< 0.0001*	38.6
Egg masses–Late nymphs	15.27	< 0.0001*	35.4
Egg masses–Adults	77.85	< 0.0001*	38.2
Early nymphs–Late nymphs	8.11	0.0044*	54.7
Early nymphs–Adults	4.47	0.034	64.6
Late nymphs–Adults	25.14	0.0001*	53.9

^a^ Critical values are adjusted for multiple comparisons using a Bonferroni correction: α/*m*, where α is the familywise critical value (0.05) and *m* is the number of comparisons (6). Thus, comparisons are considered significant when *P* < 0.0083, indicated with a *.

All life stages of *H*. *halys* were observed on 88 plant taxa, which we therefore classified as hosts. On the other hand, no life stages of *H*. *halys* were observed on 43 plant taxa, and these were classified as non-hosts. The remaining 123 cultivars were classified as partial hosts by virtue of the presence of at least one but not all life stages. Table 2 presents the 25 host plant taxa with the highest mean abundance of *H*. *halys*. Notably, maples (Family Sapindaceae) and legumes (Family Leguminosae) constituted half of these top 25 hosts. Table 3 presents the 43 non-hosts, comprised mainly of cultivars belonging to Pinaceae and Sapindaceae. A summary of the numbers of each life stage found on each plant taxon over the course of the study is available in S1 Table.

**Table 2:** Top hosts for nymph and adult *Halyomorpha halys*. Mean total abundance (± SE) of nymphs and adults per 1 min. survey (No. / Min.) for the top twenty-five plant taxa classified as hosts (~10% of all sampled taxa).

Species	Cultivar	Family	No. / Min. (± SE)
*Syringa pekinensis* (Rupr.) P.S.Green & M.C.Chang	Zhang Zhiming	Oleaceae	5.56 (± 1.02)
*Sophora japonica* (L.) Schott	Millstone	Leguminosae	4.42 (± 0.86)
*Syringa pekinensis* (Rupr.) P.S.Green & M.C.Chang	Morton	Oleaceae	3.62 (± 0.54)
*Evodia daniellii* (Benn.) T.G.Hartley		Rutaceae	3.58 (± 0.86)
*Acer x freemanii*	Jeffersred	Sapindaceae	3.30 (± 0.80)
*Acer pensylvanicum* L.		Sapindaceae	2.53 (± 0.91)
*Cercis canadensis* L.		Leguminosae	2.28 (± 0.37)
*Malus*	Mary Potter	Rosaceae	2.18 (± 0.95)
*Ulmus americana* L.	Valley Forge	Ulmaceae	2.01 (± 0.36)
*Ficus carica* L.	Chicago Hardy	Moraceae	1.93 (± 0.47)
*Acer rubrum* L.	Brandywine	Sapindaceae	1.93 (± 0.24)
*Ulmus*	Patriot	Ulmaceae	1.88 (± 0.63)
*Acer rubrum* L.	Armstrong	Sapindaceae	1.87 (± 0.24)
*Acer rubrum* L.	Bowhall	Sapindaceae	1.87 (± 0.43)
*Cladrastis kentukea* (Dum.Cours.) Rudd		Leguminosae	1.82 (± 0.24)
*Acer rubrum* L.	October Glory	Sapindaceae	1.78 (± 0.17)
*Evodia hupehensis* (Benn.) T.G.Hartley		Rutaceae	1.78 (± 0.34)
*Liquidambar styraciflua* L.		Altingiaceae	1.76 (± 0.51)
*Malus*	Donald Wyman	Rosaceae	1.72 (± 0.22)
*Sophora japonica* (L.) Schott	Regent	Leguminosae	1.65 (± 0.18)
*Koelreuteria paniculata* Laxm.		Sapindaceae	1.60 (± 0.18)
*Tilia tomentosa* Moench	Sterling	Malvaceae	1.58 (± 0.25)
*Cladrastis kentukea* (Dum.Cours.) Rudd	Perkins Pink	Leguminosae	1.49 (± 0.29)
*Acer rubrum* L.	Franksred	Sapindaceae	1.49 (± 0.13)
*Syringa reticulata* (Blume) H.Hara	Ivory Silk	Oleaceae	1.36 (± 0.47)

**Table 3:** Species and cultivars on which no *Halyomorpha halys* individuals of any life stage were observed (non-hosts).

Species	Cultivar	Family	Classification
*Abies nordmanniana* (Steven) Spach		Pinaceae	Gymnosperm
*Acer davidii* Franch.		Sapindaceae	Angiosperm
*Acer palmatum* Thunb.	Emperor I	Sapindaceae	Angiosperm
*Acer palmatum* Thunb.	Moonfire	Sapindaceae	Angiosperm
*Acer palmatum* Thunb.	Sango Kaku	Sapindaceae	Angiosperm
*Acer palmatum* var. *dissectum* Thunb.	Crimson Queen	Sapindaceae	Angiosperm
*Acer palmatum* var. *dissectum* Thunb.	Inaba Shidare	Sapindaceae	Angiosperm
*Acer palmatum* var. *dissectum* Thunb.	Seiryu	Sapindaceae	Angiosperm
*Aesculus hippocastanum* L.	Baumannii	Sapindaceae	Angiosperm
*Cedrus atlantica* (Endl.) Manetti ex Carrière	Kroh's Twisted	Pinaceae	Gymnosperm
*Cedrus deodara* (Roxb. ex D.Don) G.Don	Karl Fuchs	Pinaceae	Gymnosperm
*Cercidiphyllum japonicum* Siebold & Zucc. ex J.J.Hoffm. & J.H.Schult.bis	Red Fox	Cercidiphyllaceae	Angiosperm
*Chamaecyparis obtusa* (Siebold & Zucc.) Endl.	Aurea Nana	Cupressaceae	Gymnosperm
*Chamaecyparis obtusa* (Siebold & Zucc.) Endl.	Compacta	Cupressaceae	Gymnosperm
*Chamaecyparis obtusa* (Siebold & Zucc.) Endl.	Gimborn's Beauty	Cupressaceae	Gymnosperm
*Chamaecyparis obtusa* (Siebold & Zucc.) Endl.	Kosteri	Cupressaceae	Gymnosperm
*Cornus kousa* F.Buerger ex Hance	Radiant Rose	Cornaceae	Angiosperm
*Ginkgo biloba* L.	Saratoga	Ginkgoaceae	Gymnosperm
*Hamamelis* x *intermedia*	Jelena	Hamamelidaceae	Angiosperm
*Hamamelis* x *intermedia*	Pallida	Hamamelidaceae	Angiosperm
*Juniperus chinensis* L.	Torulosa	Cupressaceae	Gymnosperm
*Physocarpus opulifolius* (L.) Maxim.	Center Glow	Rosaceae	Angiosperm
*Picea breweriana* S.Watson		Pinaceae	Gymnosperm
*Picea koraiensis* Nakai		Pinaceae	Gymnosperm
*Picea meyeri* Rehder & E.H.Wilson		Pinaceae	Gymnosperm
*Picea omorika* (Pancic) Purk.	Pendula	Pinaceae	Gymnosperm
*Picea pungens* Engelm.	Fastigiata	Pinaceae	Gymnosperm
*Picea pungens* Engelm.	Glauca	Pinaceae	Gymnosperm
*Picea pungens* Engelm.	Glauca Iseli Fastigata	Pinaceae	Gymnosperm
*Picea pungens* Engelm.	Glauca Majestic Blue	Pinaceae	Gymnosperm
*Picea pungens* Engelm.	Hoopsii	Pinaceae	Gymnosperm
*Pinus cembra* L.	Chalet	Pinaceae	Gymnosperm
*Pinus densiflora* Siebold & Zucc.	Umbraculifera	Pinaceae	Gymnosperm
*Pinus koraiensis* Siebold & Zucc.		Pinaceae	Gymnosperm
*Pinus nigra* J.F.Arnold	Arnold Sentinel	Pinaceae	Gymnosperm
*Pinus parvifolia*		Pinaceae	Gymnosperm
*Pinus strobus* L.	Pendula	Pinaceae	Gymnosperm
*Pinus thunbergii* Parl.	Thunderhead	Pinaceae	Gymnosperm
*Prunus mume* (Siebold) Siebold & Zucc.	Bonita	Rosaceae	Angiosperm
*Prunus serrula* Franch.	Tibetica	Rosaceae	Angiosperm
*Sequoiadendron giganteum* (Lindl.) J.Buchholz		Cupressaceae	Gymnosperm
*Thuja plicata* Donn ex D.Don	Emerald Cone	Cupressaceae	Gymnosperm
*Tsuga canadensis* (L.) Carrière	Pendula	Pinaceae	Gymnosperm

### Use of angiosperms and gymnosperms

**Fig 1:**
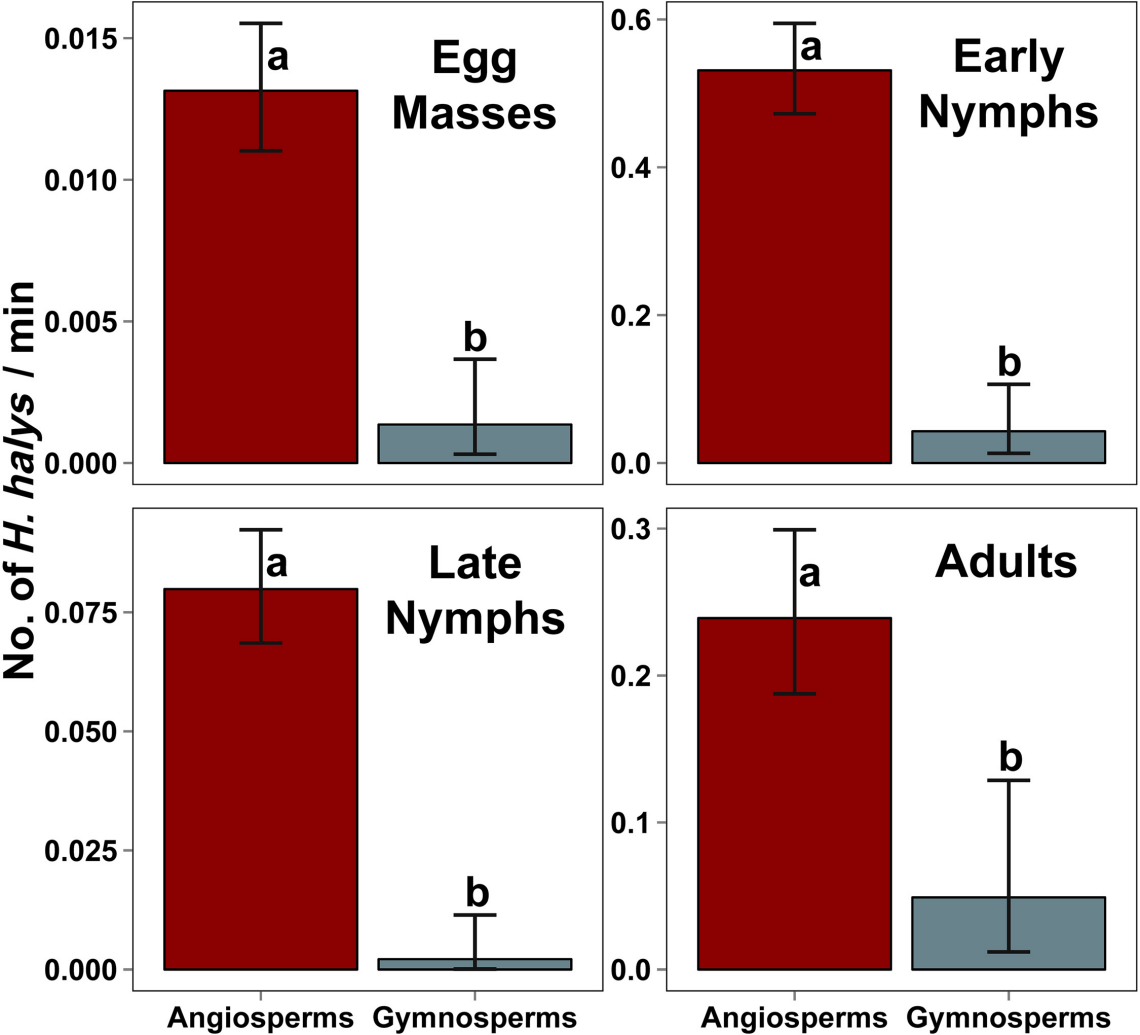
Relationship between *Halyomorpha halys* abundance and plant taxonomic status across stink bug life stages. Model estimated mean abundances (and 95% CI) are plotted for egg masses, early instar nymphs, late instar nymphs and adults. For each life stage, angiosperms supported significantly more stink bugs than gymnosperms based on Tukey’s HSD comparisons (α = 0.05) indicated by different letters above the bars.

Across all life stages, *H*. *halys* abundances were significantly higher on angiosperms than gymnosperms (Fig 1; GLMs for egg mass T _(252)_ = -3.7, P <0.001; early instar nymphs T _(252)_ = -5.4, P <0.001; late instar nymphs T _(252)_ = -3.5, P <0.001; adults T _(252)_ = -5.1, P <0.001). The abundance of egg masses, early instar nymphs, late instar nymphs and adults was respectively 10, 12, 37, and 5 times higher on angiosperms than gymnosperms.

Notably, all plant taxa classified as hosts in our study were angiosperms. On the other hand, all gymnosperms were classified as either non-hosts or partial hosts. Among the non-hosts, plant taxa in the pine family (Family Pinaceae) were the most frequently reported (20 taxa; Table 3). The genera with the corresponding number of non-host taxa (parenthetically) were: *Abies* (1), *Acer* (7), *Aesculus* (1), *Cedrus* (2), *Cercidiphyllum* (1), *Chamaecyparis* (4), *Cornus* (1), *Ginko* (1), *Hamamelis* (2), *Juniperus* (1), *Physocarpus* (1), *Picea* (9), *Pinus* (7), *Prunus* (2), *Sequoiadendron* (1), *Thuja* (1), and *Tsuga* (1).

## Discussion

By surveying 254 species and cultivated varieties of trees and shrubs under the controlled conditions of production nurseries, we were able to quantify the abundance and host plant use of *H*. *halys*. We identified 88 host taxa, 43 non-host taxa, and 123 partial hosts supporting some but not all life stages of *H*. *halys*. In doing so, we discovered variation in *H*. *halys* host use among plants that may guide the development of landscapes refractory to this stink bug.

Our results agree with previous work in North America [11,21,31], Asia [12], and Europe [24] which observed one or more life stages of *H*. *halys* on a broad range of woody plants in managed and non-managed settings. Favored hosts found in this study match those of previous ones for several genera (Table 2). *Prunus* is a genus that appears on our list of the 25 most utilized hosts and other lists of common hosts for *H*. *halys* [21,31]. Other genera found on our list of the most commonly used hosts that also appear on other host lists include *Malus*, *Syringa*, *Acer*, *Cladrastis* and *Cercis* [21,31] (see S2 Table for an explicit comparison between the present study and previous studies for plant genera that include host taxa). Lee et al. [12] noted the affinity of *H*. *halys* for hosts in the Fabaceae and Rosaceae. In addition to these families, Oleaceae, Sapindaceae, Rutaceae, Ulmaceae, Moraceae, Altingiaceae, and Malvaceae supported the greatest abundances of *H*. *halys* over three years of this study (Table 2). Because of our broad sampling, we were also able to identify host plants in nine genera that, to the best of our knowledge, have not previously been listed as host plants for *H*. *halys*: *Cercidiphyllum*, *Evodia*, *Gleditsia*, *Halesia*, *Nyssa*, *Oxydendrum*, *Parrotia*, *Pseudocydonia*, and *Styrax*.

We also identified 43 plant taxa not used at all by *H*. *halys* in our study. Interestingly, these taxa included congeners of plants we identified as hosts, underscoring strong intraspecific and intrageneric variation in *H*. *halys* abundances and host plant use. For example, *H*. *halys* was never observed on several varieties of *Acer palmatum* while most cultivars of its congener *Acer rubrum* were heavily utilized. Several cultivars of *Ginkgo biloba* supported notable numbers of *H*. *halys* nymphs and adults, whereas *Ginkgo biloba* ‘Saratoga’ supported none.

In our study, the timing or absence of fruiting resources on particular taxa likely contributed to their status as non-hosts [18]. For example, early fruiting cultivars of shrubs like *Hamamelis x intermedia* or non-fruiting trees such as male *Ginkgo biloba* ‘Saratoga’ were devoid of all life stages of *H*. *halys* and classified as non-hosts. Flowering and fruiting of *Hamamelis* occurs in winter and early spring, well in advance of the arrival of *H*. *halys* into the nursery [18,35]. In addition, some trees do not fruit abundantly until they reach maturity [47]; this could potentially lead to an underestimation of the list of plants used by the highly mobile stages. However, 10 of the 28 non-host taxa for which we have phenology data (2 angiosperms and 8 gymnosperms; data available for Year 3 only) did bear reproductive structures. Thus, plant maturity and fruiting are not the only reasons that plants are on the non-host list. In interpreting our designation of taxa as non-hosts, we urge caution for several taxa for which relatively few observations were made. For example, the number of observations of *Acer davidii* was four over the entire course of the study; due to the small number of tree visits, the placement of this species as a non-host is subject to future modification (Table 3, S1 Table). By contrast, *Acer davidii’s* congener *Acer palmatum var*. *dissectum Inaba Shidare* was observed 144 times over the three years of the study and its designation as a non-host is therefore well-supported (Table 3, S1 Table).

The patterns of intraspecific variation in host use by *H*. *halys* we report here mirrors those of other studies that demonstrate variation in host use among varieties of apples. Fujisawa [32] and Funayama [33] attributed intraspecific variation in patterns of host use to differences in fruiting times among cultivars of apples. Funayama [33] noted the importance of multiple hosts in the normal development of *H*. *halys*. Recent work by Martinson et al. [20] demonstrated the strong positive relationship between the presence of fruit and the abundance of *H*. *halys* adults on individual trees. The remarkable ability of *H*. *halys* to track quality resources in space and time has been detailed in several studies [12,14,16–18]. This explains at least in part the pattern of broad host use in *H*. *halys*, as different species and cultivars will present resources of differing quality in the nurseries throughout the growing season.

Stage specific differences in patterns of host use in this study reflect those found in previous studies of *H*. *halys* on woody plants. Stage specific shifts in host use were noted as different instars of *H*. *halys* tracked resources on different woody hosts [11]. In non-managed settings in several locations in North Carolina and Virginia, the broadest range of hosts were used by adult *H*. *halys*, the fewest hosts were used as oviposition sites, and nymphs utilized many more hosts than ovipositing females, but slightly fewer hosts than adults [31].Similarly, we found egg masses on the fewest numbers of plant taxa, whereas highly mobile adults were found on the greatest number (Table 1). Early and late instar nymphs were found on intermediate numbers of hosts.

A noteworthy finding is the difference in the use of gymnosperms by *H*. *halys* as observed in our study to that in Asian literature. The use of gymnosperms by *H*. *halys* is well established in Asia [12,32,38,48–52]. Specifically, some gymnosperms such as Japanese cedar serve as important hosts for overwintered adults early in the season [12,51]. However, our study confirms the use of several families, genera, species, and cultivars of gymnosperms as partial hosts for *H*. *halys* in North America (Table 3), but none of the gymnosperms studied were classified as hosts. Although gymnosperms are used as partial hosts by *H*. *halys*, it is noteworthy that gymnosperms supported far lower *H*. *halys* abundances than did angiosperms (Fig 1). Moreover, the list of non-hosts was dominated by gymnosperms. The Pinaceae and Cupressaceae in particular each contained several genera and species of non-hosts.

The practical implications of our study are that several species and cultivars of woody ornamental trees and shrubs presently in production do not appear to be utilized by any life stage of *H*. *halys* and by our definition, they are not hosts. By planting these varieties, landowners may enjoy lower abundances of *H*. *halys* in their landscapes, with the additional benefit of spawning fewer *H*. *halys* that would become nuisance pests as they enter homes and businesses later in autumn. Gymnosperms can help to diversify landscapes with trees that provide valuable ecosystem services including water infiltration, carbon sequestration, and as a buffer against invasive species [53]. Our findings provide evidence that gymnosperms provide a rich source of plant material refractory to *H*. *halys* for use in landscapes. We also believe that growers who produce these resistant varieties may enjoy a marketing advantage in states and countries within the invaded range of *H*. *halys*.

## Supporting Information

S1 Table The status of species and cultivars of trees and shrubs as hosts for *Halyomorpha halys* based on repeated visual surveys in two Maryland nurseries. (DOCX)

S2 Table Host utilization by *Halyomorpha halys* in our study in comparison with existing literature. (DOCX)

## References

[1] Hoebeke ER, Carter ME. *Halyomorpha halys* (Stål) (Heteroptera: Pentatomidae): a polyphagous plant pest from Asia newly detected in North America. Proc Entomol Soc Wash. 2003;105: 225–237.

[2] Northeastern IPM Center. Where is BMSB?—StopBMSB.org [Internet]. 2015 [cited 30 Aug 2015]. Available: http://www.stopbmsb.org/where-is-bmsb/

[3] Wermelinger B, Wyniger D, Forster B. First records of an invasive bug in Europe: *Halyomorpha halys* Stål (Heteroptera: Pentatomidae), a new pest on woody ornamentals and fruit trees? Mitteilungen Schweiz Entomol Ges. 2008;81: 1–8.

[4] Callot H, Brua C. *Halyomorpha halys* (Stål,1855), the marmorated stink bug, new species for the fauna of France (Heteroptera Pentatomidae). Entomol. 2013;69: 69–71.

[5] Pansa MG, Asteggiano L, Costamagna C, Vittone G, Tavella L. First discovery of *Halyomorpha halys* in peach orchards in Piedmont. Inf Agrar. 2013;69: 60–61.

[6] Heckmann R. Erster nachweis von *Halyomorpha halys* (STÅL, 1855) (Heteroptera: Pentatomidae) fur Deutschland. Heteropteron H. 2012;36: 17–18.

[7] Vétek G, Haltrich A, Rédei D. First record of the brown marmorated stink bug, *Halyomorpha halys* (Hemiptera: Heteroptera: Pentatomidae), in Hungary, with description of the genitalia of both sexes. Zootaxa. 2014;3780: 194–200. 10.11646/zootaxa.3780.1.8 24871833

[8] Milonas PG, Partsinevelos GK. First report of brown marmorated stink bug *Halyomorpha halys* Stål (Hemiptera: Pentatomidae) in Greece. EPPO Bull. 2014;44: 183–186. 10.1111/epp.12129

[9] Commonwealth of Australia. Brown marmorated stink bug: emergency measures for break bulk and containerised vehicles, machinery, automotive parts and tyres [Internet]. Mar 2015 [cited 12 Jun 2015]. Available: http://www.agriculture.gov.au/import/industry-advice/ian/15/03-2015

[10] MacLellan R. Brown marmorated stink bug: a potential risk to New Zealand. Surveillance. 2013;40: 34–36.

[11] Nielsen AL, Hamilton GC. Life history of the invasive species *Halyomorpha halys* (Hemiptera: Pentatomidae) in northeastern United States. Ann Entomol Soc Am. 2009;102: 608–616. 10.1603/008.102.0405

[12] Lee D-H, Short BD, Joseph SV, Bergh JC, Leskey TC. Review of the biology, ecology, and management of *Halyomorpha halys* (Hemiptera: Pentatomidae) in China, Japan, and the Republic of Korea. Environ Entomol. 2013;42: 627–641. 10.1603/EN13006 23905725

[13] Kuhar TP, Kamminga KL, Whalen J, Dively GP, Brust G, Hooks CRR, et al The pest potential of brown marmorated stink bug on vegetable crops. Plant Health Prog Online. 2012; 10.1094/PHP-2012-0523-01-BR

[14] Leskey TC, Hamilton GC, Nielsen AL, Polk DF, Rodriguez-Saona C, Bergh JC, et al Pest status of the brown marmorated stink bug, *Halyomorpha halys* in the USA. Outlooks Pest Manag. 2012;23: 218–226. 10.1564/23oct07

[15] Rice KB, Bergh CJ, Bergmann EJ, Biddinger DJ, Dieckhoff C, Dively G, et al Biology, ecology, and management of brown marmorated stink bug (Hemiptera: Pentatomidae). J Integr Pest Manag. 2014;5: A1–A13. 10.1603/IPM14002

[16] Venugopal PD, Coffey PL, Dively GP, Lamp WO. Adjacent habitat influence on stink bug (Hemiptera: Pentatomidae) densities and the associated damage at field corn and soybean edges. PLoS ONE. 2014;9: e109917 10.1371/journal.pone.0109917 25295593 PMC4190369

[17] Venugopal PD, Dively GP, Lamp WO. Spatiotemporal dynamics of the invasive *Halyomorpha halys* (Hemiptera: Pentatomidae) in and between adjacent corn and soybean fields. J Econ Entomol. 2015;108: 2231–2241. 10.1093/jee/tov188 26453711

[18] Martinson HM, Venugopal PD, Bergmann EJ, Shrewsbury PM, Raupp MJ. Fruit availability influences the seasonal abundance of invasive stink bugs in ornamental tree nurseries. J Pest Sci. 2015;88: 461–468. 10.1007/s10340-015-0677-8

[19] Hiruki C. Paulownia witches’-broom disease important in east Asia. Acta Hortic. 1999;496: 63–68. 10.17660/ActaHortic.1999.496.6

[20] Martinson HM, Raupp MJ, Shrewsbury PM. Invasive stink bug wounds trees, liberates sugars, and facilitates native Hymenoptera. Ann Entomol Soc Am. 2013;106: 47–52. 10.1603/AN12088

[21] Bernon G. Biology of *Halyomorpha halys*, the brown marmorated stink bug (BMSB). United States Department of Agriculture, Animal and Plant Health Inspection Service, Center for Plant Health Science and Technology; 2004 p. 17. Report No.: T3P01.

[22] Hamilton GC. Brown marmorated stink bug. Am Entomol. 2009;55: 19–20.

[23] Inkley DB. Characteristics of home invasion by the brown marmorated stink bug (Hemiptera: Pentatomidae). J Entomol Sci. 2012;47: 125–130.

[24] Haye T, Wyniger D, Gariepy TD. Recent range expansion of brown marmorated stink bug in Europe. In: Müller G, Pospichil R, Robinson W, editors. Proceedings of the Eighth International Conference on Urban Pests. Kft. Veszprém, Hungary: OOK Press; 2014. pp. 309–314.

[25] Cooper R. Brown marmorated stink bug in the urban setting: overwintering pest in residential and commercial buildings In: Leskey TC, Hamilton GC, editors. Kearneysville, WV: Northeast IPM Center; 2010 p. 15.

[26] Painter RH. Insect resistance in crop plants New York: MacMillan; 1951.

[27] Maxwell FG, Jennings PR. Breeding plants resistant to insects New York: John Wiley & Sons; 1980.

[28] Potter DA. Urban landscape pest management In: Bennett GW, Owens JM, editors. Advances in urban pest management. New York: van Nostrand Reinhold Company; 1986 pp. 219–251. Available: http://agris.fao.org/agris-search/search.do?recordID=US8743820

[29] Raupp MJ, Koehler CS, Davidson JA. Advances in implementing integrated pest management for woody landscape plants. Annu Rev Entomol. 1992;37: 561–585. 10.1146/annurev.en.37.010192.003021

[30] Herms DA. Strategies for deployment of insect resistant ornamental plants In: Wagner MR, Clancy KM, Lieutier F, Paine TD, editors. Mechanisms and deployment of resistance in trees to insects. Springer Netherlands; 2002 pp. 217–237. Available: http://link.springer.com/chapter/10.1007/0-306-47596-0_10

[31] Bakken AJ, Schoof SC, Bickerton M, Kamminga KL, Jenrette JC, Malone S, et al Occurrence of brown marmorated stink bug (Hemiptera: Pentatomidae) on wild hosts in nonmanaged woodlands and soybean fields in North Carolina and Virginia. Environ Entomol. 2015;44: 1011–1021. 10.1093/ee/nvv092 26314046

[32] Fujisawa T. Damage and control of the brown marmorated stink bug in apple orchards. Jpn Agric Technol. 2001;45: 42–47.

[33] Funayama K. Oviposition and development of *Halyomorpha halys* (Stål) and *Homalogonia obtusa* (Walker) (Heteroptera: Pentatomidae) on apple [*Molus pumila*] trees. Jpn J Appl Entomol Zool. 2002;46: 1–6.

[34] Zhang J-M, Wang H, Zhao L-X, Zhang F, Yu G-Y. Damage to an organic apple orchard by the brown marmorated stink bug, *Halyomorpha halys* and its control strategy. Chin Bull Entomol. 2007;44: 898–901.

[35] Venugopal PD, Martinson HM, Bergmann EJ, Shrewsbury PM, Raupp MJ. Edge effects influence the abundance of the invasive *Halyomorpha halys* (Hemiptera: Pentatomidae) in woody plant nurseries. Environ Entomol. 2015;44: 474–479. 10.1093/ee/nvv061 26313952

[36] Dirr MA. Manual of woody landscape plants III Champaign, IL: Stiples; 2009.

[37] The Plant List [Internet]. [cited 30 Aug 2015]. Available: http://www.theplantlist.org/

[38] Oda M, Sugiura T, Nakanishi Y, Shibata E, Uesumi Y. Ecological studies of stink bugs attacking the fruit trees, 1: the prevalence of seasonal observations by light trap, and the ecology on the occurrence of fruit trees and mulberry under field observations. Bull Nara Agric Exp Stn Jpn. 1981;11: 53–62.

[39] Oda M, Nakanishi Y, Uesumi Y. Ecological studies of stink bugs attacking fruit trees. Report 4: fluctuations in the hibernated population of the brown-marmorated stink bug, *Halyomorpha halys* (Stål) and seasonal prevalence of the adults after hibernation. Bull Nara Agric Exp Stn Jpn. 1982;13: 66–73.

[40] Velasco LRI, Walter GH. Availability of different host plant species and changing abundance of the polyphagous bug *Nezara viridula* (Hemiptera: Pentatomidae). Environ Entomol. 1992;21: 751–759.

[41] Panizzi AR. Wild hosts of Pentatomids: ecological significance and role in their pest status on crops. Annu Rev Entomol. 1997;42: 99–122. 10.1146/annurev.ento.42.1.99 15012309

[42] Ver Hoef JM, Boveng PL. Quasi-Poisson vs. negative binomial regression: How should we model overdispersed count data? Ecology. 2007;88: 2766–2772. 10.1890/07-0043.1 18051645

[43] R Development Core Team. R: A language and environment for statistical computing The R foundation for statistical computing, Vienna, Austria [Internet]. 2014 Available: http://www.R-project.org/

[44] Cayuela L, Granzow-de la Cerda Í, Albuquerque FS, Golicher DJ. taxonstand: An r package for species names standardisation in vegetation databases. Methods Ecol Evol. 2012;3: 1078–1083. 10.1111/j.2041-210X.2012.00232.x

[45] Hothorn T, Bretz F, Westfall P. Simultaneous inference in general parametric models. Biom J. 2008;50: 346–363. 10.1002/bimj.200810425 18481363

[46] Wickham H. ggplot2: elegant graphics for data analysis [Internet]. New York: Springer; 2009 Available: http://had.co.nz/ggplot2/book

[47] Marrotte EL. Why fruit trees fail to bear [Internet]. University of Connecticut; 2004 Available: http://www.ladybug.uconn.edu/factsheets/tp_05_fruittreesfailtobeal.html

[48] Kawada H, Kitamura C. Bionomics of the brown marmorated stink bug, *Halyomorpha mista*. Jpn J Appl Entomol Zool. 1983;27: 304–306.

[49] Qin W. Occurrence rule and control techniques of *Halyomorpha picus*. Plant Prot. 1990;16: 22–23.

[50] Yanagi T, Hagihara Y. Ecology of the brown marmorated stink bug. Plant Prot. 1980;34: 315–321.

[51] Funayama K. Does the brown-marmorated stink bug, *Halyomorpha halys* (Stål) (Heteroptera: Pentatomidae) reproduce by feeding on the cones of Japanese cedar, *Cryptomeria japonica* D. Don? Jpn J Appl Entomol Zool. 2005;49: 265–268.

[52] Yu G-Y, Zhang J-M. The brown marmorated stink bug, *Halyomorpha halys* (Heteroptera: Pentatomidae) in P. R. China. International Workshop on Biological Control of Invasive Species of Forests. Beijing, China; 2007. pp. 58–62.

[53] Clapp JC, Ryan HDP, Harper RW, Bloniarz DV. Rationale for the increased use of conifers as functional green infrastructure: A literature review and synthesis. Arboric J. 2014;36: 161–178. 10.1080/03071375.2014.950861

