# Supplementary material for: Host Plant Use by the Invasive Halyomorpha halys (Stål) on Woody Ornamental Trees and Shrubs
Source: PLoS One. 2016 Feb 23;11(2):e0149975. doi: 10.1371/journal.pone.0149975 (PMC4764356; doi:10.1371/journal.pone.0149975)
Supplement: S1 Table — (DOCX) [file pone.0149975.s001.docx]

**S1 Table**. **The status of species and cultivars of trees and shrubs as hosts for *Halyomorpha halys* based on repeated visual surveys in two Maryland nurseries.** The abundance of each life stage of *H. halys* is given as the summed total count across all 1 min. surveys. Host status is as follows. Hosts: each life stage observed during the study period. Partial hosts: at least one but not all life stages observed. Non-hosts: no life stages observed.

|  | **species** | **cultivar** | **family** | **classification** | **surveys** | **egg masses** | **early nymphs** | **late nymphs** | **adults** | **host status** |
| --- | --- | --- | --- | --- | --- | --- | --- | --- | --- | --- |
| 1 | *Abies koreana* E.H.Wilson |  | Pinaceae | Gymnosperm | 636 | 0 | 3 | 0 | 8 | partial |
| 2 | *Abies nordmanniana* (Steven) Spach | Ambrolauria | Pinaceae | Gymnosperm | 18 | 0 | 0 | 0 | 2 | partial |
| 3 | *Abies nordmanniana* (Steven) Spach |  | Pinaceae | Gymnosperm | 72 | 0 | 0 | 0 | 0 | non-host |
| 4 | *Acer campestre* L. | Evelyn | Sapindaceae | Angiosperm | 288 | 5 | 235 | 23 | 119 | host |
| 5 | *Acer davidii* Franch. |  | Sapindaceae | Angiosperm | 4 | 0 | 0 | 0 | 0 | non-host |
| 6 | *Acer griseum* (Franch.) Pax |  | Sapindaceae | Angiosperm | 396 | 1 | 88 | 13 | 29 | host |
| 7 | *Acer palmatum* Thunb. | Bloodgood | Sapindaceae | Angiosperm | 243 | 2 | 2 | 0 | 35 | partial |
| 8 | *Acer palmatum* Thunb. | Emperor I | Sapindaceae | Angiosperm | 72 | 0 | 0 | 0 | 0 | non-host |
| 9 | *Acer palmatum* Thunb. | Moonfire | Sapindaceae | Angiosperm | 48 | 0 | 0 | 0 | 0 | non-host |
| 10 | *Acer palmatum* Thunb. | Red Emperor | Sapindaceae | Angiosperm | 263 | 2 | 3 | 0 | 15 | partial |
| 11 | *Acer palmatum* Thunb. | Sango Kaku | Sapindaceae | Angiosperm | 96 | 0 | 0 | 0 | 0 | non-host |
| 12 | *Acer palmatum* var. *dissectum* Thunb. | Crimson Queen | Sapindaceae | Angiosperm | 24 | 0 | 0 | 0 | 0 | non-host |
| 13 | Acer palmatum var. *dissectum* Thunb. | Inaba Shidare | Sapindaceae | Angiosperm | 144 | 0 | 0 | 0 | 0 | non-host |
| 14 | *Acer palmatum* var. *dissectum* Thunb. | Seiryu | Sapindaceae | Angiosperm | 18 | 0 | 0 | 0 | 0 | non-host |
| 15 | *Acer palmatum* var. *dissectum* Thunb. | Viridis | Sapindaceae | Angiosperm | 90 | 0 | 0 | 0 | 1 | partial |
| 16 | *Acer pensylvanicum* L. |  | Sapindaceae | Angiosperm | 32 | 1 | 58 | 6 | 17 | host |
| 17 | *Acer rubrum* L. | Armstrong | Sapindaceae | Angiosperm | 282 | 5 | 366 | 154 | 6 | host |
| 18 | *Acer rubrum* L. | Bowhall | Sapindaceae | Angiosperm | 216 | 2 | 369 | 16 | 18 | host |
| 19 | *Acer rubrum* L. | Brandywine | Sapindaceae | Angiosperm | 528 | 19 | 829 | 92 | 99 | host |
| 20 | *Acer rubrum* L. | Franksred | Sapindaceae | Angiosperm | 1530 | 40 | 1829 | 299 | 145 | host |
| 21 | *Acer rubrum* L. | October Glory | Sapindaceae | Angiosperm | 960 | 22 | 1352 | 305 | 51 | host |
| 22 | *Acer rubrum* L. | Sun Valley | Sapindaceae | Angiosperm | 72 | 0 | 62 | 12 | 0 | partial |
| 23 | *Acer rufinerve* Siebold & Zucc. |  | Sapindaceae | Angiosperm | 84 | 0 | 69 | 0 | 0 | partial |
| 24 | *Acer saccharum* Marshall | Commemoration | Sapindaceae | Angiosperm | 192 | 3 | 169 | 22 | 9 | host |
| 25 | *Acer saccharum* Marshall | Green Mountain | Sapindaceae | Angiosperm | 1625 | 32 | 1401 | 240 | 163 | host |
| 26 | *Acer saccharum* Marshall | Legacy | Sapindaceae | Angiosperm | 524 | 17 | 503 | 18 | 18 | host |
| 27 | *Acer truncatum* Bunge |  | Sapindaceae | Angiosperm | 72 | 0 | 1 | 0 | 4 | partial |
| 28 | *Acer* x *freemanii* | Jeffersred | Sapindaceae | Angiosperm | 117 | 4 | 347 | 20 | 19 | host |
| 29 | *Acer* x *tegmentosum* | White Tigress | Sapindaceae | Angiosperm | 108 | 0 | 63 | 3 | 1 | partial |
| 30 | *Aesculus* *hippocastanum* L. | Baumannii | Sapindaceae | Angiosperm | 6 | 0 | 0 | 0 | 0 | non-host |
| 31 | *Aesculus* x *carnea* | Briotii | Sapindaceae | Angiosperm | 276 | 0 | 6 | 1 | 15 | partial |
| 32 | *Aesculus* x *carnea* | Fort McNair | Sapindaceae | Angiosperm | 270 | 3 | 69 | 1 | 47 | host |
| 33 | *Amelanchier* x *grandiflora* | Autumn Brilliance | Rosaceae | Angiosperm | 860 | 14 | 737 | 82 | 219 | host |
| 34 | *Amelanchier* x *grandiflora* | Princess Diana | Rosaceae | Angiosperm | 300 | 2 | 64 | 20 | 42 | host |
| 35 | *Betula nigra* L. | BNMTF | Betulaceae | Angiosperm | 333 | 2 | 141 | 8 | 25 | host |
| 36 | *Betula nigra* L. | Cully | Betulaceae | Angiosperm | 84 | 2 | 11 | 0 | 3 | partial |
| 37 | *Betula nigra* L. | Heritage | Betulaceae | Angiosperm | 84 | 2 | 35 | 1 | 0 | partial |
| 38 | *Betula papyrifera* Marshall | Renci | Betulaceae | Angiosperm | 138 | 1 | 68 | 2 | 29 | host |
| 39 | *Calocedrus decurrens* (Torr.) Florin |  | Cupressaceae | Angiosperm | 120 | 0 | 3 | 0 | 8 | partial |
| 40 | *Carpinus betulus* L. | Fastigiata | Betulaceae | Angiosperm | 770 | 10 | 451 | 17 | 30 | host |
| 41 | *Carpinus betulus* L. | Frans Fontaine | Betulaceae | Angiosperm | 228 | 0 | 27 | 1 | 31 | partial |
| 42 | *Carya illinoinensis* (Wangenh.) K.Koch | Choctaw | Juglandaceae | Angiosperm | 48 | 0 | 19 | 4 | 66 | partial |
| 43 | *Cedrus atlantica* (Endl.) Manetti ex Carrière | Glauca | Pinaceae | Gymnosperm | 234 | 0 | 0 | 0 | 2 | partial |
| 44 | *Cedrus atlantica* (Endl.) Manetti ex Carrière | Kroh's Twisted | Pinaceae | Gymnosperm | 75 | 0 | 0 | 0 | 0 | non-host |
| 45 | *Cedrus deodara* (Roxb. ex D.Don) G.Don | Karl Fuchs | Pinaceae | Gymnosperm | 72 | 0 | 0 | 0 | 0 | non-host |
| 46 | *Cedrus deodara* (Roxb. ex D.Don) G.Don | Shalimar | Pinaceae | Gymnosperm | 96 | 0 | 4 | 0 | 6 | partial |
| 47 | *Celtis koraiensis* Nakai |  | Cannabaceae | Angiosperm | 12 | 0 | 0 | 1 | 6 | partial |
| 48 | *Cercidiphyllum japonicum* Siebold & Zucc. ex J.J.Hoffm. & J.H.Schult.bis | Red Fox | Cercidiphyllaceae | Angiosperm | 10 | 0 | 0 | 0 | 0 | non-host |
| 49 | *Cercidiphyllum japonicum* Siebold & Zucc. ex J.J.Hoffm. & J.H.Schult.bis |  | Cercidiphyllaceae | Angiosperm | 317 | 1 | 321 | 9 | 98 | host |
| 50 | *Cercis canadensis* L. | Alba | Leguminosae | Angiosperm | 96 | 0 | 123 | 25 | 125 | partial |
| 51 | *Cercis canadensis* L. | Appalachian Red | Leguminosae | Angiosperm | 168 | 5 | 85 | 2 | 14 | host |
| 52 | *Cercis canadensis* L. | Covey | Leguminosae | Angiosperm | 162 | 3 | 88 | 10 | 53 | host |
| 53 | *Cercis canadensis* L. | Forest Pansy | Leguminosae | Angiosperm | 222 | 3 | 160 | 15 | 82 | host |
| 54 | *Cercis canadensis* L. | Pink Heartbreaker | Leguminosae | Angiosperm | 96 | 0 | 116 | 3 | 42 | partial |
| 55 | *Cercis canadensis* L. |  | Leguminosae | Angiosperm | 360 | 16 | 707 | 55 | 57 | host |
| 56 | *Chamaecyparis nootkatensis* D.Don | Pendula | Cupressaceae | Gymnosperm | 438 | 0 | 0 | 0 | 26 | partial |
| 57 | *Chamaecyparis nootkatensis* D.Don | Pendula Glauca | Cupressaceae | Gymnosperm | 66 | 0 | 1 | 0 | 0 | partial |
| 58 | *Chamaecyparis obtusa* (Siebold & Zucc.) Endl. | Aurea Nana | Cupressaceae | Gymnosperm | 45 | 0 | 0 | 0 | 0 | non-host |
| 59 | *Chamaecyparis obtusa* (Siebold & Zucc.) Endl. | Compacta | Cupressaceae | Gymnosperm | 66 | 0 | 0 | 0 | 0 | non-host |
| 60 | *Chamaecyparis obtusa* (Siebold & Zucc.) Endl. | Crippsii | Cupressaceae | Gymnosperm | 258 | 0 | 0 | 0 | 1 | partial |
| 61 | *Chamaecyparis obtusa* (Siebold & Zucc.) Endl. | Gimborn's Beauty | Cupressaceae | Gymnosperm | 27 | 0 | 0 | 0 | 0 | non-host |
| 62 | *Chamaecyparis obtusa* (Siebold & Zucc.) Endl. | Kosteri | Cupressaceae | Gymnosperm | 27 | 0 | 0 | 0 | 0 | non-host |
| 63 | *Chionanthus retusus* Lindl. & Paxton |  | Oleaceae | Angiosperm | 204 | 0 | 1 | 4 | 26 | partial |
| 64 | *Cladrastis kentukea* (Dum.Cours.) Rudd | Perkins Pink | Leguminosae | Angiosperm | 219 | 8 | 289 | 15 | 23 | host |
| 65 | *Cladrastis kentukea* (Dum.Cours.) Rudd |  | Leguminosae | Angiosperm | 450 | 17 | 570 | 105 | 145 | host |
| 66 | *Cornus controversa* Hemsl. |  | Cornaceae | Angiosperm | 162 | 0 | 2 | 17 | 48 | partial |
| 67 | *Cornus florida* L. | Appalachian Spring | Cornaceae | Angiosperm | 198 | 0 | 38 | 0 | 10 | partial |
| 68 | *Cornus florida* L. | Cherokee Princess | Cornaceae | Angiosperm | 452 | 0 | 78 | 1 | 38 | partial |
| 69 | *Cornus florida* L. | Cloud 9 | Cornaceae | Angiosperm | 186 | 0 | 22 | 6 | 11 | partial |
| 70 | *Cornus florida* L. | COMCO #1 | Cornaceae | Angiosperm | 405 | 0 | 59 | 1 | 32 | partial |
| 71 | *Cornus florida* L. | Jean's Appalachian Snow | Cornaceae | Angiosperm | 132 | 0 | 1 | 0 | 33 | partial |
| 72 | *Cornus florida* L. | Kay's Appalachian Mist | Cornaceae | Angiosperm | 132 | 0 | 3 | 0 | 12 | partial |
| 73 | *Cornus florida* L. |  | Cornaceae | Angiosperm | 12 | 0 | 3 | 0 | 9 | partial |
| 74 | *Cornus florida* x *kousa* L. | Aurora | Cornaceae | Angiosperm | 75 | 0 | 0 | 1 | 0 | partial |
| 75 | *Cornus florida* x *kousa* L. | Celestial | Cornaceae | Angiosperm | 21 | 0 | 21 | 0 | 24 | partial |
| 76 | *Cornus florida* x *kousa* L. | Constellation | Cornaceae | Angiosperm | 73 | 0 | 16 | 0 | 13 | partial |
| 77 | *Cornus florida* x *kousa* L. | Ruth Ellen | Cornaceae | Angiosperm | 36 | 0 | 22 | 0 | 4 | partial |
| 78 | *Cornus florida* x *kousa* L. | Stellar Pink | Cornaceae | Angiosperm | 212 | 1 | 30 | 1 | 21 | host |
| 79 | *Cornus kousa* F.Buerger ex Hance | Madison | Cornaceae | Angiosperm | 80 | 0 | 2 | 0 | 7 | partial |
| 80 | *Cornus kousa* F.Buerger ex Hance | National | Cornaceae | Angiosperm | 108 | 0 | 26 | 3 | 7 | partial |
| 81 | *Cornus kousa* F.Buerger ex Hance | Radiant Rose | Cornaceae | Angiosperm | 16 | 0 | 0 | 0 | 0 | non-host |
| 82 | *Cornus kousa* F.Buerger ex Hance | Santomi | Cornaceae | Angiosperm | 570 | 0 | 83 | 19 | 34 | partial |
| 83 | *Cornus kousa* var. *chinensis* | Milky Way | Cornaceae | Angiosperm | 315 | 0 | 6 | 2 | 31 | partial |
| 84 | *Cornus macrophylla* Wall. |  | Cornaceae | Angiosperm | 168 | 4 | 95 | 14 | 44 | host |
| 85 | *Cornus officinalis* Siebold & Zucc. |  | Cornaceae | Angiosperm | 252 | 0 | 4 | 3 | 31 | partial |
| 86 | *Cornus walteri* Wangerin |  | Cornaceae | Angiosperm | 84 | 0 | 30 | 0 | 15 | partial |
| 87 | *Crataegus crusgalli* L. | Cruzam | Rosaceae | Angiosperm | 12 | 0 | 0 | 0 | 1 | partial |
| 88 | *Crataegus laevigata* (Poir.) DC. | Superba | Rosaceae | Angiosperm | 135 | 1 | 24 | 2 | 7 | host |
| 89 | *Crataegus phaenopyrum* (L.f.) Medik. |  | Rosaceae | Angiosperm | 123 | 0 | 24 | 2 | 4 | partial |
| 90 | *Crataegus viridis* L. | Winter King | Rosaceae | Angiosperm | 948 | 6 | 325 | 26 | 50 | host |
| 91 | *Cryptomeria japonica* (Thunb. ex L.f.) D.Don | Black Dragon | Cupressaceae | Gymnosperm | 153 | 0 | 0 | 0 | 4 | partial |
| 92 | *Cryptomeria japonica* (Thunb. ex L.f.) D.Don | Gyokuryu | Cupressaceae | Gymnosperm | 180 | 0 | 0 | 0 | 1 | partial |
| 93 | *Cryptomeria japonica* (Thunb. ex L.f.) D.Don | Yoshino | Cupressaceae | Gymnosperm | 309 | 0 | 37 | 1 | 122 | partial |
| 94 | *Cryptomeria japonica* (Thunb. ex L.f.) D.Don |  | Cupressaceae | Gymnosperm | 72 | 0 | 21 | 1 | 3 | partial |
| 95 | *Cupressocyparis leylandii* A.B.Jacks. & Dallim. |  | Cupressaceae | Gymnosperm | 96 | 0 | 36 | 2 | 10 | partial |
| 96 | *Evodia daniellii* (Benn.) T.G.Hartley |  | Rutaceae | Angiosperm | 12 | 9 | 15 | 9 | 19 | host |
| 97 | *Evodia hupehensis* (Benn.) T.G.Hartley |  | Rutaceae | Angiosperm | 180 | 4 | 176 | 75 | 69 | host |
| 98 | *Ficus carica* L. | Chicago Hardy | Moraceae | Angiosperm | 45 | 2 | 3 | 6 | 78 | host |
| 99 | *Ginkgo biloba* L. | Autumn Gold | Ginkgoaceae | Gymnosperm | 384 | 4 | 38 | 0 | 25 | partial |
| 100 | *Ginkgo biloba* L. | Magyar | Ginkgoaceae | Gymnosperm | 240 | 0 | 0 | 0 | 19 | partial |
| 101 | *Ginkgo biloba* L. | Princeton Sentry | Ginkgoaceae | Gymnosperm | 288 | 7 | 93 | 0 | 9 | partial |
| 102 | *Ginkgo biloba* L. | Saratoga | Ginkgoaceae | Gymnosperm | 72 | 0 | 0 | 0 | 0 | non-host |
| 103 | *Gleditsia triacanthos* L. | Shademaster | Leguminosae | Angiosperm | 1248 | 13 | 589 | 155 | 469 | host |
| 104 | *Gleditsia triacanthos* L. | Skyline | Leguminosae | Angiosperm | 282 | 2 | 119 | 64 | 22 | host |
| 105 | *Halesia tetraptera* L. | Arnold Pink | Styracaceae | Angiosperm | 12 | 0 | 0 | 2 | 5 | partial |
| 106 | *Halesia tetraptera* L. |  | Styracaceae | Angiosperm | 390 | 4 | 173 | 12 | 55 | host |
| 107 | *Hamamelis* x *intermedia* | Arnold Promise | Hamamelidaceae | Angiosperm | 249 | 0 | 1 | 0 | 14 | partial |
| 108 | *Hamamelis* x *intermedia* | Diane | Hamamelidaceae | Angiosperm | 201 | 0 | 24 | 0 | 9 | partial |
| 109 | *Hamamelis* x *intermedia* | Jelena | Hamamelidaceae | Angiosperm | 42 | 0 | 0 | 0 | 0 | non-host |
| 110 | *Hamamelis* x *intermedia* | Pallida | Hamamelidaceae | Angiosperm | 12 | 0 | 0 | 0 | 0 | non-host |
| 111 | *Heptacodium miconioides* Rehder |  | Caprifoliaceae | Angiosperm | 185 | 0 | 8 | 1 | 14 | partial |
| 112 | *Hibiscus syriacus* L. | Blue Bird | Malvaceae | Angiosperm | 132 | 0 | 24 | 8 | 116 | partial |
| 113 | *Hibiscus syriacus* L. | Diana | Malvaceae | Angiosperm | 60 | 0 | 0 | 1 | 12 | partial |
| 114 | *Hibiscus syriacus* L. | Red Heart | Malvaceae | Angiosperm | 126 | 0 | 51 | 40 | 105 | partial |
| 115 | *Hibiscus syriacus* L. | Satin Blue | Malvaceae | Angiosperm | 156 | 0 | 40 | 24 | 287 | partial |
| 116 | *Hibiscus syriacus* L. | Satin Rose | Malvaceae | Angiosperm | 8 | 0 | 4 | 4 | 5 | partial |
| 117 | *Hibiscus syriacus* L. | White Chiffon | Malvaceae | Angiosperm | 4 | 0 | 1 | 0 | 0 | partial |
| 118 | *Ilex opaca* Aiton | Jersey Princess | Aquifoliaceae | Angiosperm | 84 | 0 | 10 | 1 | 1 | partial |
| 119 | *Ilex* x *aquipernyi* | Meschick | Aquifoliaceae | Angiosperm | 360 | 0 | 27 | 5 | 3 | partial |
| 120 | *Juniperus chinensis* L. | Torulosa | Cupressaceae | Gymnosperm | 72 | 0 | 0 | 0 | 0 | non-host |
| 121 | *Koelreuteria paniculata* Laxm. |  | Sapindaceae | Angiosperm | 670 | 16 | 483 | 85 | 506 | host |
| 122 | *Larix kaempferi* (Lamb.) Carrière |  | Pinaceae | Gymnosperm | 66 | 0 | 0 | 0 | 1 | partial |
| 123 | *Larix leptolepis* (Lamb.) Carrière |  | Pinaceae | Gymnosperm | 20 | 0 | 3 | 0 | 1 | partial |
| 124 | *Liquidambar styraciflua* L. | Cherokee | Altingiaceae | Angiosperm | 132 | 1 | 9 | 1 | 1 | host |
| 125 | *Liquidambar styraciflua* L. | Hapdell | Altingiaceae | Angiosperm | 29 | 0 | 0 | 0 | 17 | partial |
| 126 | *Liquidambar styraciflua* L. | Moraine | Altingiaceae | Angiosperm | 198 | 3 | 52 | 5 | 50 | host |
| 127 | *Liquidambar styraciflua* L. | Rotundiloba | Altingiaceae | Angiosperm | 96 | 3 | 29 | 0 | 3 | partial |
| 128 | *Liquidambar styraciflua* L. | Ward | Altingiaceae | Angiosperm | 48 | 1 | 1 | 14 | 1 | host |
| 129 | *Liquidambar styraciflua* L. |  | Altingiaceae | Angiosperm | 96 | 5 | 108 | 3 | 58 | host |
| 130 | *Magnolia liliiflora* x *stellata* | Ann | Magnoliaceae | Angiosperm | 84 | 0 | 3 | 0 | 6 | partial |
| 131 | *Magnolia liliiflora* x *stellata* | Merrill | Magnoliaceae | Angiosperm | 78 | 0 | 4 | 2 | 39 | partial |
| 132 | *Magnolia* x *loebneri* | Leonard Messel | Magnoliaceae | Angiosperm | 84 | 0 | 40 | 0 | 2 | partial |
| 133 | *Malus* | Adams | Rosaceae | Angiosperm | 144 | 2 | 95 | 40 | 3 | host |
| 134 | *Malus* | Donald Wyman | Rosaceae | Angiosperm | 384 | 2 | 248 | 53 | 361 | host |
| 135 | *Malus* | Mary Potter | Rosaceae | Angiosperm | 33 | 1 | 31 | 3 | 38 | host |
| 136 | *Malus* | Molten Lava | Rosaceae | Angiosperm | 300 | 3 | 137 | 15 | 245 | host |
| 137 | *Malus* | Pink Princess | Rosaceae | Angiosperm | 264 | 1 | 11 | 10 | 56 | host |
| 138 | *Malus* | Prairifire | Rosaceae | Angiosperm | 756 | 24 | 293 | 94 | 259 | host |
| 139 | *Malus* | Spring Snow | Rosaceae | Angiosperm | 72 | 0 | 34 | 0 | 0 | partial |
| 140 | *Malus baccata* (L.) Borkh. | Jackii | Rosaceae | Angiosperm | 15 | 0 | 5 | 1 | 1 | partial |
| 141 | *Malus domestica* Borkh. | Crimson Crisp | Rosaceae | Angiosperm | 72 | 0 | 2 | 3 | 98 | partial |
| 142 | *Malus domestica* Borkh. | Freedom | Rosaceae | Angiosperm | 240 | 0 | 46 | 4 | 194 | partial |
| 143 | *Malus domestica* Borkh. | Liberty | Rosaceae | Angiosperm | 354 | 1 | 17 | 12 | 192 | host |
| 144 | *Malus halliana* Koehne | Adirondack | Rosaceae | Angiosperm | 300 | 3 | 30 | 15 | 134 | host |
| 145 | *Malus sargentii* Rehder | Select A | Rosaceae | Angiosperm | 456 | 7 | 132 | 26 | 171 | host |
| 146 | *Malus* x *zumi* | Calocarpa | Rosaceae | Angiosperm | 489 | 5 | 330 | 63 | 166 | host |
| 147 | *Metasequoia glyptostroboides* Hu & W.C.Cheng |  | Cupressaceae | Gymnosperm | 177 | 0 | 43 | 1 | 30 | partial |
| 148 | *Nyssa sylvatica* Marshall | Tupelo Tower | Cornaceae | Angiosperm | 48 | 0 | 45 | 6 | 1 | partial |
| 149 | *Nyssa sylvatica* Marshall | Wildfire | Cornaceae | Angiosperm | 108 | 0 | 8 | 1 | 34 | partial |
| 150 | *Nyssa sylvatica* Marshall |  | Cornaceae | Angiosperm | 168 | 1 | 61 | 7 | 30 | host |
| 151 | *Ostrya virginiana* (Mill.) K.Koch |  | Betulaceae | Angiosperm | 72 | 0 | 11 | 1 | 2 | partial |
| 152 | *Oxydendrum arboreum* (L.) DC. |  | Ericaceae | Angiosperm | 180 | 2 | 61 | 4 | 22 | host |
| 153 | *Parrotia persica* C.A.Mey. | Ruby Vase | Hamamelidaceae | Angiosperm | 72 | 0 | 2 | 1 | 0 | partial |
| 154 | *Parrotia persica* C.A.Mey. | Vanessa | Hamamelidaceae | Angiosperm | 12 | 0 | 0 | 0 | 14 | partial |
| 155 | *Parrotia persica* C.A.Mey. |  | Hamamelidaceae | Angiosperm | 228 | 1 | 26 | 2 | 26 | host |
| 156 | *Physocarpus opulifolius* (L.) Maxim. | Center Glow | Rosaceae | Angiosperm | 12 | 0 | 0 | 0 | 0 | non-host |
| 157 | *Picea abies* (L.) H.Karst. |  | Pinaceae | Gymnosperm | 408 | 0 | 29 | 1 | 2 | partial |
| 158 | *Picea breweriana* S.Watson |  | Pinaceae | Gymnosperm | 15 | 0 | 0 | 0 | 0 | non-host |
| 159 | *Picea koraiensis* Nakai |  | Pinaceae | Gymnosperm | 15 | 0 | 0 | 0 | 0 | non-host |
| 160 | *Picea meyeri* Rehder & E.H.Wilson |  | Pinaceae | Gymnosperm | 66 | 0 | 0 | 0 | 0 | non-host |
| 161 | *Picea omorika* (Pancic) Purk. | Pendula | Pinaceae | Gymnosperm | 168 | 0 | 0 | 0 | 0 | non-host |
| 162 | *Picea omorika* (Pancic) Purk. |  | Pinaceae | Gymnosperm | 436 | 0 | 0 | 0 | 14 | partial |
| 163 | *Picea orientalis* (L.) Peterm. | Atrovirens | Pinaceae | Gymnosperm | 84 | 0 | 0 | 0 | 1 | partial |
| 164 | *Picea orientalis* (L.) Peterm. | Aurea Compacta | Pinaceae | Gymnosperm | 126 | 0 | 0 | 0 | 7 | partial |
| 165 | *Picea orientalis* (L.) Peterm. | Gracillis | Pinaceae | Gymnosperm | 60 | 0 | 0 | 0 | 1 | partial |
| 166 | *Picea pungens* Engelm. | Blue Diamond | Pinaceae | Gymnosperm | 102 | 0 | 1 | 0 | 0 | partial |
| 167 | *Picea pungens* Engelm. | Fastigiata | Pinaceae | Gymnosperm | 54 | 0 | 0 | 0 | 0 | non-host |
| 168 | *Picea pungens* Engelm. | Fat Albert | Pinaceae | Gymnosperm | 218 | 0 | 1 | 0 | 1 | partial |
| 169 | *Picea pungens* Engelm. | Glauca | Pinaceae | Gymnosperm | 16 | 0 | 0 | 0 | 0 | non-host |
| 170 | *Picea pungens* Engelm. | Glauca Fastigata | Pinaceae | Gymnosperm | 8 | 0 | 0 | 0 | 3 | partial |
| 171 | *Picea pungens* Engelm. | Glauca Iseli Fastigata | Pinaceae | Gymnosperm | 66 | 0 | 0 | 0 | 0 | non-host |
| 172 | *Picea pungens* Engelm. | Glauca Majestic Blue | Pinaceae | Gymnosperm | 78 | 0 | 0 | 0 | 0 | non-host |
| 173 | *Picea pungens* Engelm. | Glauca Van Sikes | Pinaceae | Gymnosperm | 286 | 0 | 0 | 0 | 1 | partial |
| 174 | *Picea pungens* Engelm. | Hoopsii | Pinaceae | Gymnosperm | 178 | 0 | 0 | 0 | 0 | non-host |
| 175 | *Pinus bungeana* Zucc. ex Endl. |  | Pinaceae | Gymnosperm | 84 | 0 | 0 | 0 | 1 | partial |
| 176 | *Pinus cembra* L. | Chalet | Pinaceae | Gymnosperm | 66 | 0 | 0 | 0 | 0 | non-host |
| 177 | *Pinus cembra* L. | Silver Sheen | Pinaceae | Gymnosperm | 54 | 0 | 37 | 1 | 38 | partial |
| 178 | *Pinus densiflora* Siebold & Zucc. | Umbraculifera | Pinaceae | Gymnosperm | 27 | 0 | 0 | 0 | 0 | non-host |
| 179 | *Pinus flexilis* E.James | Vanderwolf's Pyramid | Pinaceae | Gymnosperm | 234 | 0 | 0 | 0 | 22 | partial |
| 180 | *Pinus koraiensis* Siebold & Zucc. | Morris Blue | Pinaceae | Gymnosperm | 162 | 0 | 1 | 0 | 0 | partial |
| 181 | *Pinus koraiensis* Siebold & Zucc. |  | Pinaceae | Gymnosperm | 156 | 0 | 0 | 0 | 0 | non-host |
| 182 | *Pinus nigra* J.F.Arnold | Arnold Sentinel | Pinaceae | Gymnosperm | 27 | 0 | 0 | 0 | 0 | non-host |
| 183 | *Pinus parvifolia* Siebold & Zucc. |  | Pinaceae | Gymnosperm | 36 | 0 | 0 | 0 | 0 | non-host |
| 184 | *Pinus strobus* L. | Pendula | Pinaceae | Gymnosperm | 72 | 0 | 0 | 0 | 0 | non-host |
| 185 | *Pinus thunbergii* Parl. | Thunderhead | Pinaceae | Gymnosperm | 36 | 0 | 0 | 0 | 0 | non-host |
| 186 | *Pinus wallichiana* A.B.Jacks. |  | Pinaceae | Gymnosperm | 24 | 0 | 0 | 0 | 2 | partial |
| 187 | *Platanus* x *acerifolia* | Bloodgood | Platanaceae | Angiosperm | 1422 | 23 | 1296 | 67 | 298 | host |
| 188 | *Platanus* x *acerifolia* | Yarwood | Platanaceae | Angiosperm | 228 | 2 | 165 | 8 | 34 | host |
| 189 | *Prunus avium* (L.) L. | BaDa Bing | Rosaceae | Angiosperm | 90 | 0 | 10 | 1 | 35 | partial |
| 190 | *Prunus avium* (L.) L. | Stella | Rosaceae | Angiosperm | 42 | 0 | 20 | 0 | 24 | partial |
| 191 | *Prunus cerasifera* Ehrh. | Crimson Pointe | Rosaceae | Angiosperm | 54 | 0 | 0 | 2 | 32 | partial |
| 192 | *Prunus cerasifera* Ehrh. | Cripoizam | Rosaceae | Angiosperm | 288 | 2 | 25 | 11 | 80 | host |
| 193 | *Prunus cerasifera* Ehrh. | Thundercloud | Rosaceae | Angiosperm | 352 | 2 | 2 | 11 | 30 | host |
| 194 | *Prunus cerasus* L. | Montmorency | Rosaceae | Angiosperm | 48 | 0 | 23 | 5 | 12 | partial |
| 195 | *Prunus cerasus* L. |  | Rosaceae | Angiosperm | 72 | 0 | 1 | 1 | 7 | partial |
| 196 | *Prunus mume* (Siebold) Siebold & Zucc. | Bonita | Rosaceae | Angiosperm | 3 | 0 | 0 | 0 | 0 | non-host |
| 197 | *Prunus mume* (Siebold) Siebold & Zucc. |  | Rosaceae | Angiosperm | 12 | 0 | 0 | 0 | 1 | partial |
| 198 | *Prunus persica* (L.) Batsch | Red Haven | Rosaceae | Angiosperm | 258 | 0 | 44 | 12 | 354 | partial |
| 199 | *Prunus sargentii* Rehder |  | Rosaceae | Angiosperm | 330 | 4 | 17 | 11 | 19 | host |
| 200 | *Prunus serrula* Franch. | Tibetica | Rosaceae | Angiosperm | 36 | 0 | 0 | 0 | 0 | non-host |
| 201 | *Prunus serrulata* Lindl. | Kwanzan | Rosaceae | Angiosperm | 504 | 7 | 260 | 24 | 86 | host |
| 202 | *Prunus serrulata* Lindl. | Snowgoose | Rosaceae | Angiosperm | 333 | 1 | 78 | 14 | 58 | host |
| 203 | *Prunus subhirtella* Miq. | Autumnalis | Rosaceae | Angiosperm | 66 | 2 | 74 | 41 | 0 | partial |
| 204 | *Prunus subhirtella* Miq. | Pendula | Rosaceae | Angiosperm | 288 | 3 | 29 | 18 | 89 | host |
| 205 | *Prunus subhirtella* Miq. | Pisnshzam | Rosaceae | Angiosperm | 156 | 2 | 4 | 2 | 15 | host |
| 206 | *Prunus* x *incam* | Okame | Rosaceae | Angiosperm | 456 | 3 | 169 | 2 | 32 | host |
| 207 | *Prunus* x *yedoensis* |  | Rosaceae | Angiosperm | 324 | 8 | 175 | 5 | 16 | host |
| 208 | *Pseudocydonia sinensis* (Dum.Cours.) Koehne |  | Rosaceae | Angiosperm | 96 | 1 | 4 | 12 | 11 | host |
| 209 | *Pyrus betulifolia* Bunge |  | Rosaceae | Angiosperm | 72 | 1 | 5 | 1 | 8 | host |
| 210 | *Pyrus calleryana* Decne. | Cleveland Select | Rosaceae | Angiosperm | 132 | 4 | 50 | 57 | 7 | host |
| 211 | *Pyrus communis* L. | Blake's Pride | Rosaceae | Angiosperm | 42 | 0 | 0 | 0 | 11 | partial |
| 212 | *Pyrus communis* L. | Sunrise | Rosaceae | Angiosperm | 48 | 0 | 4 | 0 | 14 | partial |
| 213 | *Pyrus fauriei* C.K.Schneid. | Westwood | Rosaceae | Angiosperm | 48 | 1 | 20 | 0 | 3 | partial |
| 214 | *Quercus acutissima* Carruth. |  | Fagaceae | Angiosperm | 213 | 3 | 104 | 20 | 7 | host |
| 215 | *Quercus alba* L. |  | Fagaceae | Angiosperm | 168 | 1 | 56 | 1 | 30 | host |
| 216 | *Quercus bicolor* Willd. |  | Fagaceae | Angiosperm | 66 | 0 | 13 | 1 | 1 | partial |
| 217 | *Quercus coccinea* Münchh. |  | Fagaceae | Angiosperm | 426 | 1 | 50 | 4 | 31 | host |
| 218 | *Quercus palustrus* Münchh. | Green Pillar | Fagaceae | Angiosperm | 132 | 2 | 19 | 0 | 1 | partial |
| 219 | *Quercus robur* L. | Fastigiata | Fagaceae | Angiosperm | 72 | 4 | 26 | 1 | 2 | host |
| 220 | *Quercus robur* L. | Regal Prince | Fagaceae | Angiosperm | 404 | 12 | 317 | 3 | 49 | host |
| 221 | *Quercus rubra* L. |  | Fagaceae | Angiosperm | 726 | 6 | 339 | 4 | 84 | host |
| 222 | *Rhus typhina* L. | Bailtiger | Anacardiaceae | Angiosperm | 96 | 0 | 0 | 0 | 7 | partial |
| 223 | *Sambucus nigra* L. | Eva | Adoxaceae | Angiosperm | 138 | 0 | 0 | 0 | 1 | partial |
| 224 | *Sequoiadendron giganteum* (Lindl.) J.Buchholz |  | Cupressaceae | Gymnosperm | 3 | 0 | 0 | 0 | 0 | non-host |
| 225 | *Sophora japonica* (L.) Schott | Millstone | Leguminosae | Angiosperm | 72 | 2 | 201 | 45 | 72 | host |
| 226 | *Sophora japonica* (L.) Schott | Regent | Leguminosae | Angiosperm | 591 | 10 | 574 | 96 | 305 | host |
| 227 | *Stewartia* *pseudocamellia* var. *koreana* (Nakai ex Rehder) Sealy |  | Theaceae | Angiosperm | 157 | 0 | 3 | 0 | 31 | partial |
| 228 | *Stewartia pseudocamellia* Maxim. |  | Theaceae | Angiosperm | 272 | 2 | 23 | 3 | 67 | host |
| 229 | *Styrax japonicus* Siebold & Zucc. |  | Styracaceae | Angiosperm | 642 | 2 | 78 | 15 | 24 | host |
| 230 | *Styrax obassia* Siebold & Zucc. |  | Styracaceae | Angiosperm | 72 | 0 | 12 | 3 | 2 | partial |
| 231 | *Syringa pekinensis* (Rupr.) P.S.Green & M.C.Chang | Morton | Oleaceae | Angiosperm | 324 | 4 | 96 | 21 | 1057 | host |
| 232 | *Syringa pekinensis* (Rupr.) P.S.Green & M.C.Chang | Zhang Zhiming | Oleaceae | Angiosperm | 117 | 1 | 38 | 24 | 589 | host |
| 233 | *Syringa reticulata* (Blume) H.Hara | Ivory Silk | Oleaceae | Angiosperm | 72 | 1 | 82 | 9 | 7 | host |
| 234 | *Taxus* x *media* | Hatfeldii | Taxaceae | Gymnosperm | 12 | 0 | 0 | 0 | 10 | partial |
| 235 | *Taxus* x *media* | Hicksii | Taxaceae | Gymnosperm | 78 | 0 | 0 | 0 | 2 | partial |
| 236 | *Thuja* *occidentalis* L. | Smaragd | Cupressaceae | Gymnosperm | 144 | 0 | 9 | 5 | 0 | partial |
| 237 | *Thuja plicata* Donn ex D.Don | Atrovirens | Cupressaceae | Gymnosperm | 105 | 1 | 0 | 0 | 3 | partial |
| 238 | *Thuja plicata* Donn ex D.Don | Emerald Cone | Cupressaceae | Gymnosperm | 84 | 0 | 0 | 0 | 0 | non-host |
| 239 | *Thuja plicata* Donn ex D.Don | Zebrina | Cupressaceae | Gymnosperm | 78 | 0 | 0 | 0 | 2 | partial |
| 240 | *Thuja standishii* x *plicata* (Gordon) Carrière | Green Giant | Cupressaceae | Gymnosperm | 663 | 0 | 22 | 7 | 53 | partial |
| 241 | *Tilia americana* L. | Redmond | Malvaceae | Angiosperm | 132 | 0 | 1 | 3 | 0 | partial |
| 242 | *Tilia cordata* Mill. | Greenspire | Malvaceae | Angiosperm | 904 | 21 | 365 | 34 | 48 | host |
| 243 | *Tilia tomentosa* Moench | Sterling | Malvaceae | Angiosperm | 495 | 22 | 674 | 38 | 70 | host |
| 244 | *Tsuga canadensis* (L.) Carrière | Pendula | Pinaceae | Gymnosperm | 36 | 0 | 0 | 0 | 0 | non-host |
| 245 | *Ulmus* | Patriot | Ulmaceae | Angiosperm | 78 | 1 | 111 | 30 | 6 | host |
| 246 | *Ulmus americana* L. | Princeton | Ulmaceae | Angiosperm | 564 | 12 | 442 | 38 | 111 | host |
| 247 | *Ulmus americana* L. | Valley Forge | Ulmaceae | Angiosperm | 210 | 11 | 308 | 14 | 100 | host |
| 248 | *Ulmus parvifolia* Jacq. | Dynasty | Ulmaceae | Angiosperm | 174 | 0 | 60 | 33 | 3 | partial |
| 249 | *Ulmus parvifolia* Jacq. | Emer I | Ulmaceae | Angiosperm | 72 | 0 | 16 | 0 | 4 | partial |
| 250 | *Ulmus parvifolia* Jacq. | Emer II | Ulmaceae | Angiosperm | 431 | 2 | 151 | 11 | 35 | host |
| 251 | *Viburnum carlesii* Hemsl. | Cayuga | Adoxaceae | Angiosperm | 12 | 0 | 0 | 0 | 1 | partial |
| 252 | *Xanthoceras sorbifolia* Bunge |  | Sapindaceae | Angiosperm | 232 | 0 | 28 | 6 | 17 | partial |
| 253 | *Zelkova serrata* (Thunb.) Makino | Green Vase | Ulmaceae | Angiosperm | 297 | 4 | 32 | 38 | 2 | host |
| 254 | *Zelkova serrata* (Thunb.) Makino | Village Green | Ulmaceae | Angiosperm | 882 | 15 | 254 | 126 | 18 | host |
