# Supplementary material for: Host Plant Use by the Invasive Halyomorpha halys (Stål) on Woody Ornamental Trees and Shrubs
Source: PLoS One. 2016 Feb 23;11(2):e0149975. doi: 10.1371/journal.pone.0149975 (PMC4764356; doi:10.1371/journal.pone.0149975)
Supplement: S2 Table — (DOCX) [file pone.0149975.s002.docx]

**S2 Table. Host utilization by *Halyomorpha halys* in our study in comparison with existing literature.** These include host use by *H. halys* in Asia (Lee et al. [11]), North America (Bernon [12], Nielsen and Hamilton [13], and Bakken et al. [14]) and Europe (Haye et al. [15]).

| **Genera common to previous studies and our present study** | **Genera of woody plants utilized by *H. halys* listed in previous works but not sampled in our study** |
| --- | --- |
| *Acer* | *Ailanthus* |
| *Aesculus* | *Aralia* |
| *Amelanchier* | *Aronia* |
| *Betula* | *Asimina* |
| *Carpinus* | *Buddleia* |
| *Carya* | *Camelli* |
| *Cedrus* | *Campsis* |
| *Celtis* | *Caragana* |
| *Cercis* | *Castanea* |
| *Cornus* | *Catalpa* |
| *Cladrastis* | *Celastrus* |
| *Crataegus* | *Cephalanthus* |
| *Cryptomeria* | *Chaenomeles* |
| *Cupressus* | *Cinnamomum* |
| *Ficus* | *Citrus* |
| *Ginko* | *Clerodendrum* |
| *Hamamelis* | *Corylus* |
| *Hibiscus* | *Cotoneaster* |
| *Ilex* | *Decaisnea* |
| *Koelreutaria* | *Diospyros* |
| *Liquidambar* | *Elaeagnus* |
| *Magnolia* | *Euonymus* |
| *Malus* | *Forsythia* |
| *Platanus* | *Fraxinus* |
| *Prunus* | *Juglans* |
| *Pyrus* | *Lagerstroemia* |
| *Quercus* | *Laurus* |
| *Rhus* | *Ligustrum* |
| *Pyrus* | *Liriodendron* |
| *Sambucus* | *Lonicera* |
| *Sophora* | *Mimosa* |
| *Stewartia* | *Morus* |
| *Syringa* | *Nerium* |
| *Tilia* | *Olea* |
| *Ulmus* | *Paulownia* |
| *Viburnum* | *Populus* |
| *Zelkova* | *Platycladus* |
|  | *Punica* |
|  | *Pyracnatha* |
|  | *Rhus* |
|  | *Robinia* |
|  | *Rosa* |
|  | *Salix* |
|  | *Sassafras* |
|  | *Sequoia* |
|  | *Spiraea* |
|  | *Sorbus* |
|  | *Toona* |
|  | *Trachycarpus* |
|  | *Vitex* |
|  | *Vitis* |
|  | *Weigela* |
|  | *Wisteria* |
|  | *Ziziphus* |

**References**

11. Lee D-H, Short BD, Joseph SV, Bergh JC, Leskey TC. Review of the biology, ecology, and management of *Halyomorpha halys* (Hemiptera: Pentatomidae) in China, Japan, and the Republic of Korea. Environ Entomol. 2013;42: 627–641. doi:10.1603/EN13006

12. Bernon G. Biology of *Halyomorpha halys*, the brown marmorated stink bug (BMSB). United States Department of Agriculture, Animal and Plant Health Inspection Service, Center for Plant Health Science and Technology; 2004 p. 17. Report No.: T3P01.

13. Nielsen AL, Hamilton GC. Life history of the invasive species *Halyomorpha halys* (Hemiptera: Pentatomidae) in northeastern United States. Ann Entomol Soc Am. 2009;102: 608–616. doi:10.1603/008.102.0405

14. Bakken AJ, Schoof SC, Bickerton M, Kamminga KL, Jenrette JC, Malone S, et al. Occurrence of brown marmorated stink bug (Hemiptera: Pentatomidae) on wild hosts in nonmanaged woodlands and soybean fields in North Carolina and Virginia. Environ Entomol. in press; nvv092. doi:10.1093/ee/nvv092

15. Haye T, Wyniger D, Gariepy TD. Recent range expansion of brown marmorated stink bug in Europe. In: Müller G, Pospichil R, Robinson W, editors. Proceedings of the Eighth International Conference on Urban Pests. Kft. Veszprém, Hungary: OOK Press; 2014. pp. 309–314.
